# The relationship between lower limb muscle strength and lower extremity function in HIV disease

**DOI:** 10.4102/sajp.v73i1.360

**Published:** 2017-09-26

**Authors:** Peter C. Mhariwa, Hellen Myezwa, Mary L. Galantino, Douglas Maleka

**Affiliations:** 1 Department of Physiotherapy, Faculty of Health Sciences, University of the Witwatersrand, South Africa; 2 Stockton University Physical Therapy Program, Galloway, New Jersey, United States; 3 School of Medicine – CCEB, University of Pennsylvania, United States

## Abstract

**Background:**

Human immunodeficiency virus (HIV) negatively impacts muscle strength and function. This study aimed to establish the relationship between lower limb muscle strength and lower extremity function in HIV disease.

**Method:**

A cross-sectional study was undertaken with a sample of 113 HIV-positive participants. Lower limb muscle strength and self-reported function were established using dynamometry and the Lower Extremity Functional Scale (LEFS), respectively. Muscle strength and functional status were established in a subset of 30 HIV-negative participants to determine normative values.

**Results:**

Muscle strength for participants with HIV ranged from an ankle dorsiflexion mean of 9.33 kg/m^2^ to 15.79 kg/m^2^ in hip extensors. In the HIV-negative group, ankle dorsiflexors recorded 11.17 kg/m^2^, whereas hip extensors were the strongest, generating 17.68 kg/m^2^. In the HIV-positive group, linear regression showed a positive relationship between lower limb muscle strength and lower extremity function (*r* = 0.71, *p* = 0.00). Fifty per cent of the changes in lower extremity function were attributable to lower limb muscle strength. A simple linear regression model showed that lower limb ankle plantar flexors contributed the most to lower extremity function in this cohort, contrary to the literature which states that hip and trunk muscles are the most active in lower limb functional activities.

**Conclusion:**

Lower extremity strength impacts perceived function in individuals stabilised on antiretroviral therapy for HIV disease. These findings demonstrate that ankle plantar flexors produce more force over hip flexors. Careful attention should be paid to the implications for strength training in this population.

## Introduction

Studies have shown that human immunodeficiency virus (HIV) is associated with muscle weakness (Arenas-Pinto et al. 2016; Raso et al. 2013, 2014; Rees et al. 2016; Richert et al. 2011) but its specific influence on self-perception of lower extremity function is not known. Muscle weakness has been attributed directly to HIV disease or side effects from highly active antiretroviral therapy (HAART) on tissue oxygen extraction and utilisation (Carnethon, Gulati & Greenland 2005). As such, HIV and HAART negatively influence oxygen kinetics, limiting the extraction and use of oxygen in lower extremity musculature (Cade et al. 2003). Lower oxygen use affects physical fitness and individuals’ motivation to perform routine activities (Mendes et al. 2013). The acute phase of HIV is frequently marked by a substantial loss of physical fitness because of impairments, in particular muscle strength compromise (Raso et al. 2013).

The International Classification of Function (ICF) is an overarching theme in HIV disease management developed by the World Health Organization (2001). So, understanding impairments, functional limitations and participation restrictions associated with HIV is a prerequisite to rehabilitation intervention (Myezwa et al. 2009, 2011). Studies have shown proximal weakness in chronic HIV disease, quantified through dynamometry (Myezwa et al. 2009). In another study, one in two adults with undetectable viral load had poor lower limb muscle performance, which may place this population at risk for frailty (Neto et al. 2016), falls and fracture (Richert et al. 2011). However, the specific effect of HIV disease and muscle strength on self-report of lower extremity function is not known.

The possible causes of poor lower limb muscle performance include hypogonadism, malnutrition, anorexia, infection (Grinspoon & Mulligan 2003) and muscle wasting (Goletz et al. 1997). Wasting syndrome compromises balance in 30% of people living with HIV (Carter 2011). Each year of infection with HIV increases the risk of poor lower extremity performance by 8% (Carter 2011). Therefore, HIV is known to decrease physical activity levels and functional independence (Kinsey 2007). Function of the lower extremity includes walking on level and rough surfaces, going up and down stairs, standing, kneeling, squatting, sitting and rising from a sitting position (Rogers & Irrgang 2003).

Physical activity and functional independence are affected by several muscle disorders reported in patients who are HIV-positive on HAART (Fillipas et al. 2010; Robinson‐Papp & Simpson 2009). These disorders range in severity from myalgia and asymptomatic elevated creatine kinase (CK) to rhabdomyolysis. Although these pathologies are rare, the most common is HIV-associated myopathy. In spite of the known relationships of HIV infection and lower extremity function, little research illuminates the nuanced presentation of function in relation to muscle strength and patients’ self-perception.

The aim of this study was to establish the relationship between lower limb muscle strength and self-perceived lower extremity function in individuals stabilised on HAART for HIV disease management. Muscle strength in this study group was compared to normative muscle strength in individuals who were HIV-negative. No values for an African population could be found in the literature which necessitated the measurement of muscle strength in an HIV-negative group. Comparing an HIV-positive and an HIV-negative group may contribute to comparison of muscle strength and its relationship to function

## Methods

### Sampling strategy and size

The sample frame included 3500 patients diagnosed with HIV disease registered at Mutare Provincial Hospital in Zimbabwe. An estimate of 10% prevalence of lower extremity weakness was made based on the literature (Oursler et al. 2009) with the worst acceptable prevalence being 15% at a 95% confidence interval.

A sample size of 113 participants was calculated using standard power analysis. Systematic sampling was undertaken by choosing every third patient who met the inclusion criteria as they entered the clinic (Castillo & Daoudi 2009). The HIV-negative group was identified through the National Blood Transfusion Register of persons who undergo regular HIV tests and donate blood routinely. In a similar fashion, HIV-negative participants with an equal number of men and women were targeted. Four to six patients were included per day for 2 months until the sample size was achieved.

### Procedure

#### Patient-reported outcome measure

In patients with musculoskeletal disorders, the Lower Extremity Functional Scale (LEFS) is reliable (*r* = 0.94) (Watson et al. 2005) and construct validity is supported by comparison with the SF-36 and so has good psychometric properties (Binkley et al. 1999). This scale has been used in a population of patients with and without HIV distal sensory polyneuropathy, tested for utility in the clinical setting and was used in this study (Galantino et al. 2014). The LEFS is a questionnaire containing 20 questions about a person’s ability to perform daily activities. The LEFS can be used by clinicians as a measure of patients’ initial function, ongoing progress and outcome and to establish functional goals. It can be used to evaluate the functional impairment of a patient with a disorder of one or both lower extremities and can monitor the patient over time to evaluate the effectiveness of an intervention (Binkley et al. 1999). The first author administered and collected the LEFS on all eligible participants. A back-translated version of the Shona LEFS and an English version were offered.

#### Dynamometry

In dynamometry, resistance applied at the end of the tester range is termed a ‘break test’, whereas resistance applied throughout the range is termed a ‘make test’, and the results of the strength testing differ depending on the method used (Dutton 2005). The isometric hold (break test) shows the muscle to have a higher test grade than the resistance given throughout the range (make test), and the handheld devices used in dynamometry quantify the ‘breaking force’ necessary to depress a limb held in a specific position by the patient (Dutton 2005).

#### Leg strength measure

A handheld dynamometer incorporating a load cell and digital display set to read force in Newton or kg/m² (Bohannon 1997) was used. Lower limb muscle strength testing was performed by isolating a particular muscle or muscle group, and then an external force was applied (Dutton 2005). The dynamometer tests consisted of isometric ‘make contractions’ in which the patients for each tested muscle group pushed maximally against the plate and the piston of the handheld dynamometer for 5 s. The first author manually stabilised the body parts proximal to the tested limb segment during testing. Before testing each muscle group, the first author demonstrated the muscle contraction to be performed to the patient. A retest of muscle groups was performed 10–15 min after the initial test. The participant then held the maximal contraction for 5 s and then relaxed. The scale was read, and the score recorded to the nearest kilogram. The piston of the dynamometer was held perpendicular to the limb segment towards which it was directed, and the plate of the dynamometer was always placed in the same position on the tested limb.

### Data analysis

The statistical package for social sciences (SPSS) version 21 was used to analyse demographic data and muscle strength using means, standard deviations and frequencies. Further analysis via Wilcoxon Rank Sum test was used for comparison of lower limb muscle strength in both groups. Using multiple regression analysis (*p* value set at < 0.05), we established the relationship between lower limb muscle strength and lower extremity functioning and adjusted for demographic variables.

### Ethical considerations

Ethical clearance was obtained from the University of the Witwatersrand Human Research Ethics Committee (M120715), the Medical Research Council of Zimbabwe (MRCZ/B/514) and Mutare Provincial Hospital Research Committee. Participants signed informed consent in either English or Shona per patient preference. Individuals were included if they were adherent to highly active antiretroviral therapy (HAART), were 18–70 years old and were ambulant.

## Results

Table 1 describes the demographic characteristics of study participants. Seventy per cent (*n* = 79) of participants who were stable on HAART for HIV disease ranged between 40 and 69 years of age, whereas 80% of HIV-negative participants were < 40 years of age (see Table 1).

**TABLE 1:** Age and gender characteristics and their relationship to functional status for HIV-negative and HIV-positive groups (*n* = 143).

Demographic characteristics	HIV-negative (*n* = 30)		HIV-positive (*n* = 113)		Mean functional score	Odds ratio	95% CI	*p*
	*N*	%	*N*	%				
Gender								
Female	13	43.33	56.00	49.50	69.68	0.95	0.92–0.99	0.01*
Male	17	56.65	57.00	50.40	75.39	1.05	1.01–1.09	0.01*
Age (years)								
15–39	24	80.00	34.00	30.09	72.56	N/A	63.40–81.37	0.97
40–69	6	20.00	79.00	69.91	-	-	-	-
Employment status								
Employed	24	80.00	73.00	64.60	75.53	1.09	1.01–1.14	0.00*
Unemployed	6	20.00	29.00	25.60	62.57	0.07	0.72–1.05	0.00*
Retired	0	0.00	11.00	9.73	74.25	1.07	0.99–1.15	0.00*
Students	-	-	-	-	71.00	1.04	0.96–1.12	0.00*
Gross monthly income								
< R500	6	29.00	36.00	31.80	52.17	N/A	0.92–1.03	1.89
R501–R1000	1.0	3.33	14.00	12.30	77.06	N/A	0.94–1.06	1.72
R1001–R2000	2.0	10.00	28.00	24.70	74.10	N/A	0.98–1.08	1.69
R2001–R5001	17	56.67	33.00	29.20	16.19	N/A	0.92–1.04	1.78
> R5000	2.0	10.00	2.00	1.77	80.00	N/A	0.97–1.03	1.36
Housing environment								
Share a space or shack	0	0.00	24.00	21.20	72.67	1.00	0.96–1.04	0.23
RDP house or hostel or flat	11	36.67	20.00	17.70	80.00	1.01	1.05–1.15	0.23
Town	18	60.00	34.00	30.00	73.11	1.02	0.97–1.06	0.23
Rural	1	3.33	35.00	30.90	53.43	0.92	0.95–1.02	0.23
Transport means								
Walking	18	60.00	89.00	78.70	66.86	0.92	0.88–0.97	0.07
Wheeled	12	40.00	24.00	21.20	76.19	1.09	1.04–1.14	0.00*

*Source*: Authors’ own work

* , significant *p* ≤ 0.05.

Multiple regression conducted on the HIV-positive cohort yielded an *R* value of 0.71, *R*^2^ value of 0.50, an adjusted *R*^2^ of 0.46 and a standard error of the estimate 8.90 (*p* = 0.001) with correlation coefficient (*r* = 0.71) between lower limb muscle strength and function in participants with HIV. The coefficient of determination (0.50) indicates that 50% of lower limb function in this HIV-positive cohort is a result of lower limb muscle strength. Other variables (like mode of transport and employment status) contribute to the remaining lower extremity function in this cohort. Fifty-one per cent of the HIV-positive cohort (*n* = 73) were employed earning a salary of R2001–R5000. No regression analysis was performed on the HIV-negative cohort; however, the two groups were assessed for differences in muscle strength, and there were significant differences noted (see Table 2).

**TABLE 2:** Lower limb muscle strength kg/m^2^ in HIV-positive participants on highly active antiretroviral therapy (*n* = 113) and HIV-negative participants (*n* = 30).

Muscle group	HIV-negative (*n* = 30)						HIV-positive (*n* = 113)					
	Whole group		Male		Female		Whole group		Male		Female	
	Mean	SD	Mean	SD	Mean	SD	Mean	SD	Mean	SD	Mean	SD
Ankle plantar flexors	12.46	2.79	13.7	2.64	11.21	2.37	15.10	3.29	16.58	3.68	13.77	2.77
Ankle dorsiflexors	9.33	2.51	10.07	2.38	8.62	2.55	11.17	2.42	11.88	2.20	10.37	2.64
Knee flexors	11.81	3.63	13.2	3.62	10.27	3.02	12.65	2.24	13.79	3.76	11.35	2.17
Knee extensors	15.36	5.4	17.64	5.3	12.64	4.04	17.07	4.46	18.87	3.55	14.75	4.36
Hip flexors	11.24	3.58	13.09	3.04	9.38	3.3	13.05	3.88	15.34	3.57	10.07	2.62
Hip extensors	15.79	5.07	18.39	4.56	13.11	4.16	17.68	4.65	19.81	3.30	15.06	4.16
Hip abductors	11.51	3.12	12.88	2.77	10.11	2.85	16.32	4.72	18.19	4.58	14.35	4.72
Hip adductors	11.09	3.64	13.54	8.8	9.43	2.99	14.13	4.36	16.00	3.47	11.64	4.79

*Source*: Authors’ own work

The results for the HIV-positive and HIV-negative groups were compared for differences in strength. Overall, HIV-negative participants showed higher mean values for all lower extremity muscle strength compared to HIV-positive participants. Lower limb muscle strength was significantly different in all muscle groups for the HIV-positive group except knee flexors and extensors with *p*-values of 0.46 and 0.08, respectively (see Table 3).

**TABLE 3:** Comparison of 30 HIV-negative and 30 HIV-positive participants on highly active antiretroviral therapy (matched groups) lower limb muscle strength.

Muscle strength	HIV-positive	HIV-negative	*p*
	Mean muscle strength (kg/m^2^)	Mean muscle strength (kg/m^2^)	
Ankle plantar flexors	12.76	15.36	0.0001*
Ankle dorsiflexors	9.66	11.23	0.0001*
Knee flexors	12.30	12.73	0.4600
Knee extensors	15.72	17.08	0.0800
Hip flexors	11.67	13.33	0.0001*
Hip extensors	15.75	17.75	0.0001*
Hip abductors	11.98	16.53	0.0001*
Hip adductors	11.71	14.11	0.0001*

*Source*: Authors’ own work

* , significant *p* ≤ 0.05.

Spearman’s rho correlation established the order in which each muscle group from most to least contributed towards lower extremity functional activities; ankle plantar flexors contributed the most activity when performing lower extremity functional activities in the HIV cohort, whereas knee extensors are the least active. Knee flexors were the second, whereas hip flexors are the third most active muscle group in lower extremity function (see Table 4).

**TABLE 4:** Order of contribution of muscle groups to lower limb function in HIV-positive cohort.

Independent variables	Dependent variables	
	Functional status	Correlations ranked
Ankle plantar flexors	0.56	1
Ankle dorsi-flexors	0.45	7
Knee flexors	0.54	2
Knee extensors	0.40	8
Hip flexors	0.54	3
Hip extensors	0.46	6
Hip abductors	0.51	4
Hip adductors	0.50	5

*Source*: Authors’ own work

### Lower extremity function in HIV-positive participants on highly active antiretroviral therapy

The majority 78% (*n* = 88) had no difficulty, 15% (*n* = 17) had a little difficulty, 5% (*n* = 6) had difficulty and only 2% (*n* = 2) of participants had quite a bit of difficulty with self-report lower limb functional activities. In terms of function, male participants were functionally better than female participants. Employed participants with HIV disease who walked as a means of transportation and those with town or residential housing environments had a better self-reported functional score than those who did not.

## Discussion

To our knowledge, this is the first study carried out in Mutare, Zimbabwe, a poorly resourced environment, to establish the relationship between lower limb muscle strength and self-reported lower extremity function in individuals receiving HAART for HIV disease. In some of the participants, the LEFS was uniquely administered in Shona, a local vernacular language.

While confirming the phenomenon that strength and function are related (Erlandson et al. 2013), this study adds to the nuanced understanding of muscle changes and function that are peculiar to HIV. Fifty per cent of lower extremity function was a result of lower limb muscle strength and furthermore the order of contribution to lower limb function of which gait and locomotion are fundamental. The understanding of the specific activation of lower extremity muscles has implications for the therapists’ approach in mitigating the impact of the HIV ageing process. Reduction of muscle mass associated with ageing seems to be the primary factor responsible for reduction in lower limb muscle strength and the consequent loss of lower extremity function in elderly people (Deschenes 2004). It is estimated that ageing is associated with 20% – 40% of the decrease in muscle strength and the consequent lower extremity functional loss at 70–80 years of age and still greater reductions (50%) at 90 years of age in both sexes (Deschenes 2004). The majority of participants in our study were over 40 years of age, and, therefore, ageing could be an influencing factor with regard to the strength values obtained, with generally lower values of muscle strength in the HIV-positive group compared to the HIV-negative group.

A detailed regression model showed lower limb ankle plantar flexors contributed the most to lower extremity function in the HIV-positive cohort. This is contrary to the literature which shows that hip flexors are the dominant factor in lower extremity functional activities in a normal population (Fotoohabadi, Tully & Galea 2010). Lower extremity functional activities depend on an individual’s ability to perform sit to stand. Transitioning from a sitting to standing position is a coordinated sagittal movement between the hips, knees, lumbar and thoracic spine. Hip flexion is the dominant factor (90%) in the pre lift-off phase. Adequate trunk movements which represent proximal stability must be sufficient to propel the upper body mass forward (Riley et al. 1991). This trunk movement must be limited to prevent the possibility of falling forward at the lift-off phase (Mourey et al. 2000). Our study results note predominantly proximal weakness which may compromise the ability to perform sit to stand, consequently negatively affecting lower extremity functional activities.

Proximal weakness was found in this HIV-positive cohort with plantar flexors being the most active muscle group instead of hip and trunk muscles which is consistent with Myezwa et al.’s (2009) findings. Myezwa et al. found that HIV disease affects general function because of proximal weakness.

The order of contribution by each lower limb muscle group was established through this investigation. To our knowledge, no previous studies have examined the relationship between lower limb muscle strength and self-reported lower extremity function in people living with HIV disease. The implication for nuanced rehabilitation is potentially important for effective results of any rehabilitation intervention for living long and well with HIV disease. Studies show that individuals with HIV disease are at a greater risk for sarcopenia, an indicator of frailty, even following adjustment for age and body mass index (BMI) (Erlandson et al. 2013; Neto et al. 2016). Frailty, however, appears to be reversible in HIV through implementation of an exercise regimen (Rees et al. 2016). Consideration for a comprehensive fitness programme, including progressive resistance exercise, is prudent in optimising strength and functional outcomes (Mkandla, Myezwa & Musenge 2016; Neto et al. 2015; Shephard 2015).

## Conclusion

This study contributes to the existing body of knowledge related specifically to activation of muscle strength and impact on self-reported function in people living with HIV disease. Further research is needed to explore longitudinal changes in muscle strength and function over time and their influence on function, work and participation.
